# Automated and Multiplexed Soft Lithography for the Production of Low-Density DNA Microarrays

**DOI:** 10.3390/microarrays5040025

**Published:** 2016-09-26

**Authors:** Julie Fredonnet, Julie Foncy, Jean-Christophe Cau, Childérick Séverac, Jean Marie François, Emmanuelle Trévisiol

**Affiliations:** 1 ITAV, Université de Toulouse, CNRS, UPS, Toulouse 31000, France; julie.fredonnet@toulouse.inra.fr (J.Fre.); julie.foncy@laas.fr (J.Fon.); childerick.severac@itav.fr (C.S.); fran_jm@insa-toulouse.fr (J.M.F.); emmanuelle.trevisiol@laas.fr (E.T.); 2 LISBP, Université de Toulouse, INSA, UPS, INP, LISBP, 135 Avenue de Rangueil, Toulouse F-31077, France; 3 Innospys SAS, 3, allée des Vignes, Carbonne 31390, France; jc-cau@innopsys.fr; 4 Dendris SAS, 335 Rue du Chêne Vert, Labège 31670, France; 5 CNRS, LAAS, 7 Avenue du Colonel Roche, F-31400 Toulouse, France; 6 LAAS, Univ de Toulouse, F-31400 Toulouse, France

**Keywords:** soft lithography, microcontact printing, automation, dedicated microarrays, hybridization, multiplexing

## Abstract

Microarrays are established research tools for genotyping, expression profiling, or molecular diagnostics in which DNA molecules are precisely addressed to the surface of a solid support. This study assesses the fabrication of low-density oligonucleotide arrays using an automated microcontact printing device, the InnoStamp 40^®^. This automate allows a multiplexed deposition of oligoprobes on a functionalized surface by the use of a MacroStamp^TM^ bearing 64 individual pillars each mounted with 50 circular micropatterns (spots) of 160 µm diameter at 320 µm pitch. Reliability and reuse of the MacroStamp^TM^ were shown to be fast and robust by a simple washing step in 96% ethanol. The low-density microarrays printed on either epoxysilane or dendrimer-functionalized slides (DendriSlides) showed excellent hybridization response with complementary sequences at unusual low probe and target concentrations, since the actual probe density immobilized by this technology was at least 10-fold lower than with the conventional mechanical spotting. In addition, we found a comparable hybridization response in terms of fluorescence intensity between spotted and printed oligoarrays with a 1 nM complementary target by using a 50-fold lower probe concentration to produce the oligoarrays by the microcontact printing method. Taken together, our results lend support to the potential development of this multiplexed microcontact printing technology employing soft lithography as an alternative, cost-competitive tool for fabrication of low-density DNA microarrays.

## 1. Introduction

Immobilization of nucleic acids in array format requires the attachment of DNA molecules (probes) at well-defined positions onto the surface of a solid support. The array is then hybridized using labeled nucleic acids (DNA or RNA targets) and the matched sequences are identified by position and fluorescent signal intensity. Microarrays are established research tools for genotyping, expression profiling, or molecular diagnostics [1,2]. Different types of patterning methods can be used to fabricate microarrays [3,4,5,6,7] and the production parameters must be adjusted for the desired applications, including the degree of multiplexing, the achievable feature shape, size and pitch, the surface area that can be patterned, and the throughput of the patterning process.

A common approach to produce DNA microarrays is to mechanically spot pre-synthesized DNA probes onto a solid support using metal pins [8] or microactuated nozzles [9]. Another way to immobilize oligonucleotides on the surface is in situ high-density DNA microarray fabrication, which is based on the step by step direct synthesis of oligonucleotides on the surface using light-activated chemistry combined with photolithographic techniques using pre-made masks [10,11] or moving mirrors, leading to maskless array synthesis and flexibility in array design and manufacturing [7]. Inkjet printing process could also be used to produce long length 60-mer DNA microarrays by in situ synthesis, one nucleotide at a time [12,13]. All of these technological platforms of production have their own advantages and weaknesses [14] and, until now, the use of DNA microarrays as routine tools in analytical/clinical laboratories for rapid molecular in vitro diagnostics is still limited.

In parallel to these technologies, microcontact printing (µCP) emerged in the literature as an alternative way of deposition of self-assembled monolayers of molecules on a surface [15,16]. This technology is also termed “soft lithography” because it is based on the use of a soft elastomeric stamp, usually made of polydimethysiloxane (PDMS), which is topographically structured by casting a PDMS prepolymer against a silicon mold leading to a structured PDMS stamp. The stamp is then inked with the molecules of interest, blown dry under a stream of nitrogen, and deposited on a solid surface, leading to patterns of molecules that are defined by the topographical structures of the stamp. In this way, microcontact printing offers a simple and low-cost surface patterning methodology with high versatility and sub-micrometer accuracy. This methodology may encompass a large range of possibilities in the patterning of various biomolecules, from nucleic acids to carbohydrate molecules [17,18,19] or proteins, being today the biomolecule of choice to be microcontact printed [20,21,22,23].

The surface of a reticulated untreated PDMS stamp is covered with methyl residues and, consequently, is hydrophobic with a water contact angle of 108° [24]. This property may weaken both inking and transfer efficiency during microcontact printing of biomolecules. Since nucleic acids are negatively-charged polyelectrolytes, electrostatic interactions play a major role in determining adsorption of the nucleic acids to the stamp (inking step) and the transfer of the inked stamp to the surface of the solid support (patterning step). To promote both steps, Lange et al. [4] functionalized the reticulated PDMS stamp and glass slide surfaces with aminosilane, rendering them attractive to DNA by electrostatic reversible binding. Using a similar double-surface functionalization strategy, Xu et al. [25] designed amphiphatic DNA (so-called DNA-surfactant) by attaching a large hydrophobic group to the 3′- or 5′-end of an oligonucleotide, thus forming a molecule able to electrostatically interact with an amino-modified reticulated PDMS stamp and able to be transferred via its hydrophobic moiety to the surface of interest. Rozkiewicz et al. [26] reported the modification of reticulated PDMS stamps with positively-charged dendrimers that can attract nucleic acids and transfer them from the surface of the stamp to a target solid support. All of these processes require the chemical functionalization of the reticulated PDMS stamp surface and are not straightforward. Thibault et al. [27] showed that, contrary to expectation, unmodified reticulated PDMS stamp surface can retain nucleic acids which can then be transferred onto the substrate surface, enabling a facile route for the production of DNA microarrays. To account for this patterning process using unmodified reticulated PDMS surfaces, these authors showed that DNA transferred onto the target surface originated from nucleic acids retained by low molecular weight (LMW) siloxane fragments present within and at the surface of the cured PDMS stamp [28].

Although various biomolecules have been successfully microcontact printed, the production of biomolecule-arrays by microcontact printing remains a challenging task and needs an effective, fast, robust, and low-cost automation process. In this paper, we explored the potential of producing an oligonucleotide array using an automated microcontact printing device, the InnoStamp 40^®^, and compared biochips fabricated by this technology with those using a conventional microarrayer. Due to the difference in patterning (i.e., printing DNA under dry conditions versus spotting a biomolecule in solution) we showed that probe and target concentrations, as well as the nature of the surface chemistry onto which the printing is made, strongly impact on the quality and robustness of the printed biochips. Taking into account these parameters, this automated soft-lithography technology can be foreseen as an alternative competitive tool for the fabrication of low-density DNA microarrays.

## 2. Materials and Methods

### 2.1. InnoStamp 40^®^ for Multiplexed Printing of Biomolecules Using MacroStamp^TM^

**Figure 1:**
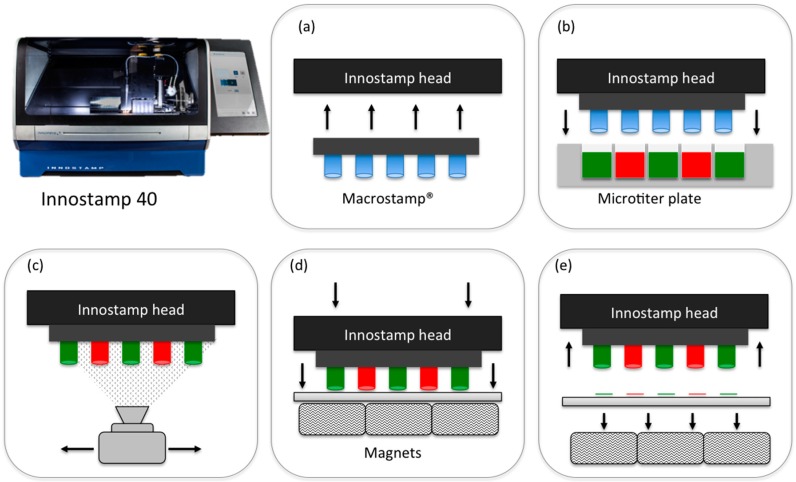
Printing of different molecules (green/red) in one step using InnoStamp 40^®^ and MacroStamp^TM^. (**a**) Loading of the patterned magnetic MacroStamp^TM^; (**b**) inking; (**c**) drying; (**d**) magnetically-assisted contact of the MacroStamp^TM^ and glass slide allowing the transfer of inked molecules; and (**e**) unloading.

**Figure 2:**
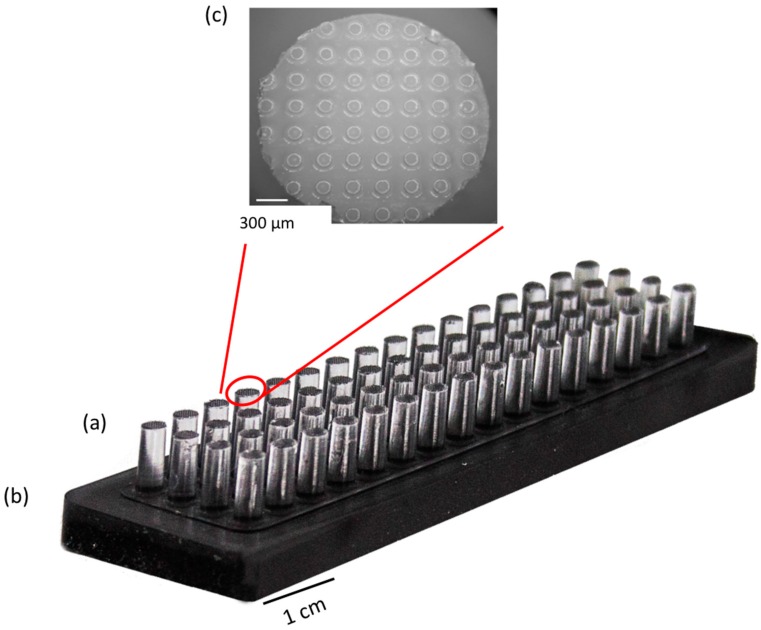
View of a 64 micropatterned pillars reticulated magnetic MacroStamp^TM^. (**a**) Micropatterned PDMS pillars; (**b**) MacroStamp^TM^ magnetic moiety containing PDMS mixed with iron powder; (**c**) zoom on the extremity of one pillar consisting of a matrix of circular patterns (50 spots per pillar, 160 µm in diameter, 300 µm pitch).

InnoStamp 40^®^ (Figure 1) is a fully automated microcontact printer (Innopsys, Carbonne, France), which assembles different modules corresponding to every step of the microcontact printing process. It utilizes magnetic fields generated by a set of magnets (NdFeB magnets, 25 mm × 25 mm × 12 mm) to manipulate magnetized PDMS stamps, allowing a homogeneous printing of oligonucleotides. The entire microcontact printing cycle consists of:

- Loading of the magnetic MacroStamp^TM^ (Figure 2) and aligning with the microtiter plate;
- Inking (1 min) by immersion of the magnetic MacroStamp^TM^ in the microtiter plate containing oligonucleotides to be deposited at the appropriate concentrations;
- Drying (30 s) of the MacroStamp^TM^ using a turbine (100 UA);
- Printing of oligonucleotides (1 min): MacroStamp^TM^ is automatically aligned and brought into contact with glass slides allowing the transfer of oligonucleotides and the one step microarray fabrication.

MacroStamp^TM^ is then removed from the surface and unloaded. It was finally cleaned by immersion in 96% ethanol (5 min) and then dried under a stream of nitrogen before storage or reuse.

### 2.2. Preparation of the Magnetic MacroStamp^TM^

Magnetic microstructured PDMS MacroStamp^TM^ (25 mm × 75 mm, Figure 2) were obtained by molding PDMS (Sylgard^®^ 184, Dow Corning, Seneffe, Belgium) on a silicon-etched mold generated by UV photolithography and reactive ion etching (RIE) with a targeted etch depth of 160 µm. The mold is silanized using octodecyltrichlorosilane (OTS) to facilitate the MacroStamp^TM^ unmolding [29]. PDMS prepolymer solution (10:1 base to curing agent ratio) was then poured on the silicon mold and partially cured at 60 °C for 15 min. Then, a mix of PDMS prepolymer solution and iron powder (Sigma Aldrich (Saint Quentin Fallavier, France), hydrogen reduced, 50 µm diameter, 50/50, *w*/*w*) is prepared by successively mixing (2 min) and degassing (5 min) the resulting magnetic PDMS prepolymer solution. These steps were repeated twice and the magnetic PDMS was then molded on the first partially-cured PDMS layer. The two layers were then reticulated at 60 °C for 4 h. The resulting MacroStamp^TM^ containing 64 microstructured (50 circular patterns, 160 µM diameter at 300 µM pitch) millimetric pillars (Figure 2) were then removed from the mold. Each MacroStamp^TM^ was cleaned before and after its use by immersion in 96% ethanol (5 min).

### 2.3. Fabrication and Processing of the Microarrays

Epoxide functionalized glass slides “Nexterion^®^ slide E” were purchased from Schott Nexterion (Jena, Germany), “Corning^®^ UltraGAPS™” amino-modified slides from Sigma-Aldrich (Saint Quentin Fallavier, France), and aldehyde-coated DendriSlides [30] from Dendris (Labège, France). HPLC purified oligonucleotide probes and targets were purchased from Sigma-Aldrich (Saint Quentin Fallavier, France) and synthesized by standard phosphoramidite method. Oligonucleotide-probes were functionalized at their 5′ extremity by an amine function via a 6-carbon alkyl spacer. Sequences used: 24-mer fluorescent probe: 5′(AmC_6_T)-ATACTCCGGGAAACTGACATCTAG-(Cy3 or Cy5)-3′; 20-mer probe: 5′(AmC_6_T)-AATATGTTTCCGGTCGTCTC-3′; complementarily-labeled 20-mer target: 5′(Cy3)-GAGACGACCGGAAACATATT-3′. Oligonucleotide-probes were diluted in phosphate buffer (Na_2_HPO_4_ 300 mM, pH 9 for Nexterion^®^ slide E, and DendriSlides and pH 7.4 for Corning^®^ UltraGAPS™) before printing (InnoStamp 40^®^) or spotting (Q-Array Mini, Genetix, Paris, France).

Unless otherwise stated, the probes were printed or spotted at concentrations ranging from 0.01 µM to 50 µM using InnoStamp 40^®^ or conventional Q-Array Mini microarrayer equipped with metal pins (SMP3, ArrayIt, EBN, Dolembeux, Belgium) delivering around 2 nL of oligonucleotide solutions per spot with a medium size diameter of 160 µm at 300 µM pitch. The printing process using the Macrostamp^TM^ make use of the magnetic force to control the pressure applied during contact to ensure a homogeneous transfer of the DNA from the PDMS onto the surface. The quality of the printing was optimal at 45% relative humidity and constant temperature of 20 °C. After oligonucleotide deposition (printing or spotting), the slides were dried overnight. DendriSlides were processed as described earlier (reduction of unstable imine function between amino-probes and aldehyde surface to stable amino function using NaBH_4,_ 3.5 mg/mL in milliQ water [30], whereas Corning^®^ UltraGAPS™ were UV cross-linked (Stratalinker^®^ UV crosslinker, 254 nm, 600 mJ/cm^2^). Epoxysilane slides did not require further processing.

### 2.4. Hybridization

Hybridizations were carried out by adding the complementary labeled target at concentrations ranging from 0.01 to 100 nM in saline-sodium phosphate buffer (5× SSC (saline-sodium citrate): 75 mM sodium citrate, 750 mM NaCl) containing sodium dodecyl sulfate (SDS 0.1%, *w*/*v*), pH 7.4, overnight at 37 °C. After hybridization, slides were washed 2 × 3 min in 2× SSC, SDS 0.2%, and 3 min in 0.1× SSC. Finally, they were dried under a stream of nitrogen.

### 2.5. Data Acquisition and Statistical Analysis

All slides were analyzed using InnoScan 710 (Innopsys, Carbonne, France), which simultaneously scans slides at two wavelengths (PMT 532 nm and 635 nm: 100%). Images were captured using Mapix software (Innopsys, Carbonne, France). Circle grids were aligned on each spot for each image. The fluorescence intensity of each spot was automatically calculated as the median of fluorescence signal minus the background signal. Fluorescence intensity of 100 spots deposited by microarrayer or printed by InnoStamp 40^®^ were analyzed for each condition. When probes were printed, the fluorescence signals of the 25 best-defined circular patterns on 4 independent millimetric pillars, located at different positions on the microarray, were collected. For each condition the mean of fluorescence intensities was calculated. The coefficient of variation (CV) was calculated as the quotient of the standard deviation to the mean of fluorescence intensity.

## 3. Results

### 3.1. General Features of the InnoStamp 40^®^ for Automated and Multiplexed Microcontact Printing

InnoStamp 40^®^ utilizes a magnetic field to manipulate magnetized polydimethylsiloxane (PDMS) stamps and to homogeneously transfer molecules on functionalized glass slides. When located on the automated head, the magnetic field is used to load and unload the magnetic MacroStamp^TM^ throughout the entire microcontact printing process (loading, inking, drying, aligning, printing, cleaning, and unloading) as depicted in Figure 1. Once inked on the micropatterned magnetic MacroStamp^TM^, the oligonucleotides were transferred on a solid surface of interest after applying a precise and homogeneous force. The magnetic field was generated from the bottom of the printing area by a set of magnets ensuring an accurate transfer of inked patterns [31].

The force applied on the MacroStamp^TM^ during the printing step was found to be 11.58 N (±0.03 N) and corresponds to a pressure of 7.9 kPa applied on the MacroStamp^TM^ and 61.0 kPa on each pillar. This value allowed a well-defined contact of the microstructured pillars on the glass slide surface without roof collapsing of the stamp.

The fabrication of a low-density DNA array was then assessed using a magnetic MacroStamp^TM^ composed of 64 pillars (4 × 16) with a diameter of 2.2 mm and pitch of 4.5 mm (Figure 2).

**Figure 3:**
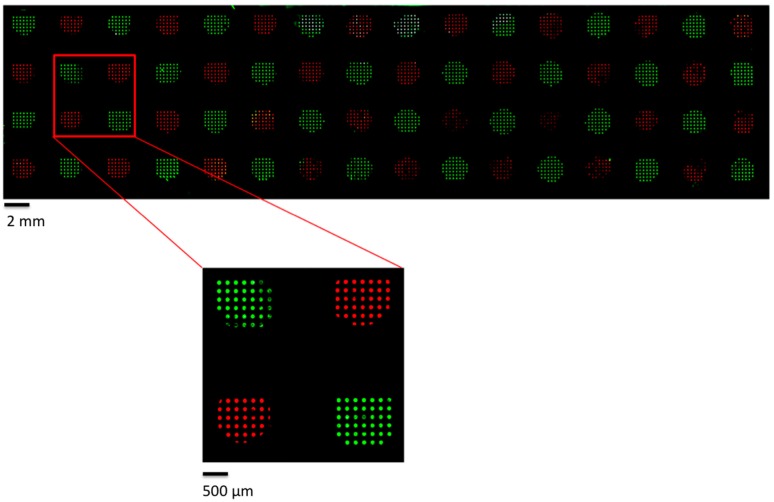
Fluorescence image of multiplexed DNA microcontact-printed using InnoStamp 40^®^ and a magnetic MacroStamp^TM^ on an epoxysilane glass slide (Green: Cy3-oligonucleotide, red: Cy5-oligonucleotide).

Each pillar contains at its extremity a repetition of 50 circular micropatterns of 160 µm diameter that are spaced from each other by 320 µm. The distance between each microstructured pillar corresponds to the one between each well of a 384-well microtiter plate, allowing the deposition of 64 different molecules in one step. These 64 pillars were inked simultaneously in a microtiter plate alternatively containing Cy5 and Cy3-labeled oligonucleotides, and the MacroStamp^TM^ was aligned and brought into contact with an epoxysilane glass slide. Figure 3 shows the resulting image obtained after scanning with InnoScan 710.

The fluorescence intensity of the printed oligonucleotides was obtained by integrating the median fluorescence signal of the individual spots on each pillar. There is no contamination between the pillars after multiplexed printing of oligonucleotide probes. An enlargement of the image shows the high resolution of the micropatterns, nicely identifying more than 25 circular patterns (spots) within each pillar validating the multiplexed deposition of oligonucleotides by microcontact printing.

### 3.2. MacroStamp^TM^ Reusability and Reliability

**Figure 4:**
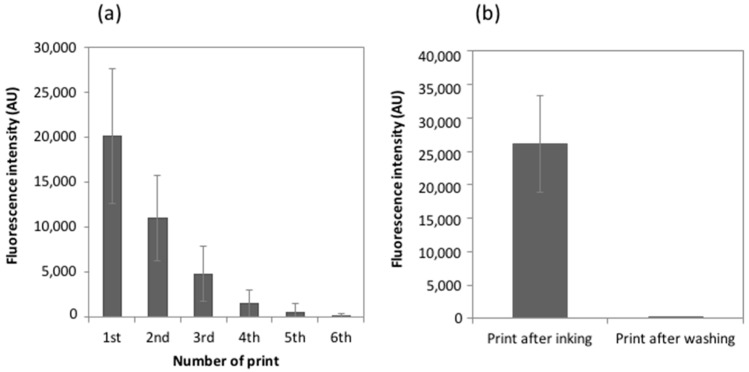
(**a**) Fluorescence intensities recorded immediately after deposition of labelled DNA probes (5 μM). One MacroStamp^TM^ was used to successively print oligonucleotides six times on DendriSlides; and (**b**) fluorescence intensities recorded after deposition of labeled DNA probes (5 μM) and after washing by ethanol. Means of 100 spots from four independent pillars for each condition are shown.

To evaluate the ability of the MacroStamp^TM^ to replicate the same arrays without re-inking, a doubly-functionalized 5′-amino-3′-Cy3 oligonucleotide inked on the stamp was printed on dendrimer-activated glass slides, as described previously, and the process was repeated six times. As shown in Figure 4a, the trend of the fluorescence intensity recorded on 100 spots from four independent pillars followed a quasi-linear decrease with respect to the number of prints, the fluorescence intensity signal almost decreasing by two-fold after each print. Therefore, this rapid decay in fluorescence prohibits a reuse of the MacroStamp^TM^ without re-inking because it shall likely produce unreliably printed slides.

**Figure 5:**
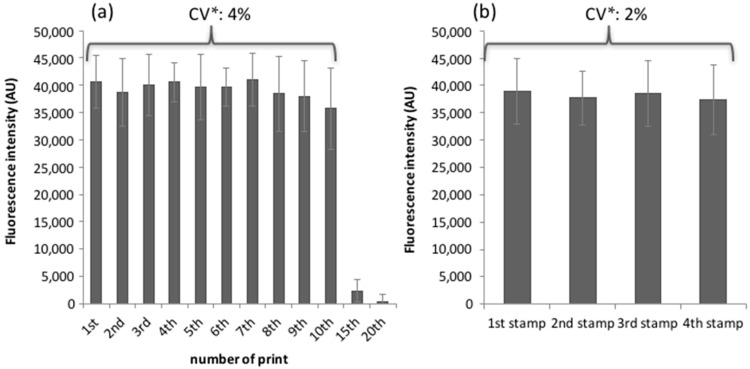
Fluorescence intensities recorded immediately after deposition of labeled oligonucleotides (5 μM). (**a**) One unique MacroStamp^TM^ was used 20 times on 20 DendriSlides. The MacroStamp^TM^ was washed by incubation in 96% ethanol (5 min) between each prints; and (**b**) Four independent MacroStamps^TM^ were used to print DNA probes on DendriSlides. Means of 100 spots from four independent pillars for each condition are shown. *CV: coefficient of variation

The second step was to implement an efficient washing procedure to remove oligonucleotide probes from the PDMS surface, in order to reuse the MacroStamp^TM^. Results determined on 100 circular micropatterns gathered from four independent pillars show that a single wash of the stamp during 1 min in 96% ethanol was sufficient to entirely remove the molecules inked on the stamp since the fluorescence signal of the doubly 5′-amino-3′-Cy3 oligonucleotides printed on the arrays dropped to the background level after this single washing step (Figure 4b). This washing step is, thus, very simple and efficient and opens the question of how many times a MacroStamp^TM^ can be reused without loss of its transfer capacities. Consequently, we proceeded to repeat the cycle which includes loading of the MacroStamp^TM^ in InnoStamp 40^®^, inking, drying, printing, unloading, washing in 96% ethanol solution, and drying before recording the fluorescent signal of 100 spots after each cycle. As reported on Figure 5a, the MacroStamp^TM^ could be washed and re-inked up to 10 times without loss of its transfer properties and the variation of the printing process over these 10 cycles was in the range of 4%.

After these 10 reuses of the MacroStamp^TM^, we observed a drastic rupture in the histograms of fluorescence intensities, which was obtained from several independent stamps. This result indicates that the surface of the PDMS was unable to load and/or transfer oligonucleotides probably because the immersion in 96% ethanol solution finally purged the PDMS of its low molecular weight siloxane-helping transfer fragments [28,32]. We also investigated the inter-stamp variability and found a consistent reliability in the printing of labeled oligonucleotides among MacroStamp^TM^ prepared from four independent batches (Figure 5b).

### 3.3. Immobilization Efficiency

**Figure 6:**
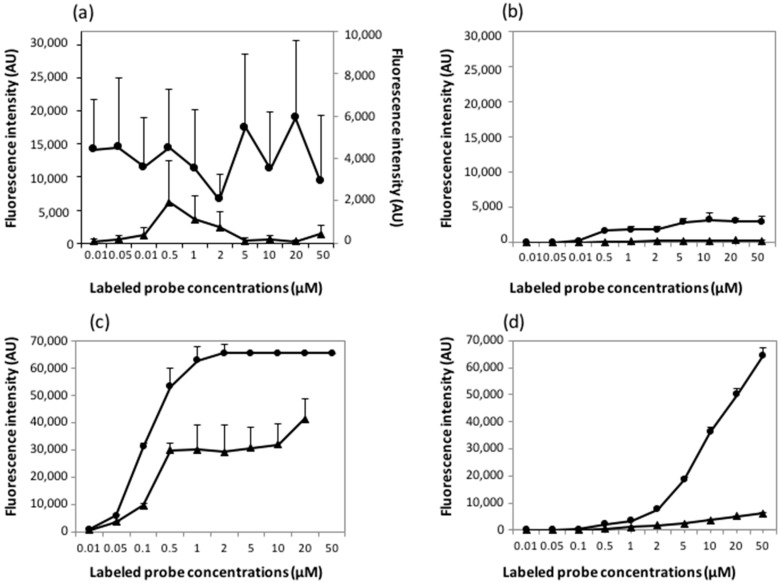
Fluorescence intensities recorded immediately after deposition (circle) or after overnight incubation in hybridization buffer without complementary target (hybridization mime, triangle). Probes were printed by InnoStamp 40^®^ on (**a**) epoxysilane slides (after hybridization mime, triangle, data have to be read using the right *y*-axis) and (**b**) amino-modified slide. Probes were spotted by contact microarrayer on (**c**) epoxysilane slides and (**d**) amino-modified slides.

Immobilization efficiency was investigated by deposition of doubly-functionalized 5′-amino-3′-Cy5-oligonucleotides by microcontact printing (InnoStamp 40^®^) or spotting (Q-Array Mini microarrayer) at increasing concentrations ranging from 0.01–50 µM on either epoxysilane glass slides (Nexterion E) or amino-modified (UltraGAPS™) slides. We chose these two types of chemically-activated supports because the first one allows direct covalent bonding of the 5′NH_2_-modified oligonucleotides by incubation of the printed slides in a chamber at room temperature and set at 45% humidity, whereas they are immobilized by electrostatic interactions on the amino-modified slides. We did not use DendriSlides in this experiment because the covalent linkage of the 5′NH_2_/3′Cy5-labeled oligonucleotides on the aldehyde functions of the DendriSlides required a reductive reaction that destroys the fluorescent dye [33]. To learn about the probe immobilization efficiency and the actual amount of probes potentially accessible to the targets on glass slides for both printing and spotting technologies, we performed the hybridization step in the absence of the complementary target. Results are reported in Figure 6.

The amount of probes printed on epoxysilane by the InnoStamp 40^®^ with the MacroStamp^TM^ was almost the same whatever the initial concentration of probes (Figure 6a). A similar behavior occurred using amino-modified slides, except that no probe was retained on this type of chemically-activated slides when printed at a concentration lower than 0.5 µM (Figure 6b) and, furthermore, the amount of retained probes on amino-modified slides was at least 10-fold lower than on epoxysilane slides. In contrast, the fluorescence intensities increased with probe concentrations using the mechanical spotting method and reached intensity saturation (~60,000 AU) at a probe concentration of 0.5 µM with the epoxysilane glass slide (Figure 6c), whereas this saturation was reached only at the highest probe concentration of 50 µM on the amino-modified slide (Figure 6d). These results indicated that more probes are deposited by the spotting technology than by the microcontact printing method. On the other hand, they also illustrated the higher capacity of the epoxysilane slides to immobilize oligonucleotide probes.

Printed and spotted oligonucleotide arrays were left resting for 12 h at room temperature before being incubated at 37 °C overnight in the hybridization buffer without targets. As shown in Figure 6, a notable effect of this hybridization-mimicking step was a drastic decrease in the fluorescence intensities over the entire probe concentration range, which was significantly more important for oligoarrays produced by microcontact printing than for those produced by conventional spotting. In quantitative terms, we found that the maximal fluorescence intensity obtained on printed oligoarrays was around 100- to 1000-fold lower than on spotted oligoarrays. Moreover, this drop in probes density was more severe with amino-modified slides than with epoxysilane slides whatever the deposition technology. This data confirms that a surface chemistry permitting covalent linkage of the biomolecules is favoring high probe immobilization efficiency at low concentration of deposited probes [34,35]. We also noticed that the diminution of fluorescence intensity versus probes concentration on epoxysilane slides by the InnoStamp 40^®^ technology followed a bell-shaped curve, with a maximum at the probe concentration of 0.5–1 µM. Such behavior is unclear, as it was not observed in the case of amino-modified slides printed similarly. Taking these results together, we can conclude that the microcontact printing technology results in lower immobilization efficiency than the mechanical spotting method. In addition, we confirmed that the covalent linkage is more appropriate than the electrostatic interaction to immobilize the oligonucleotide probes on a glass surface and, hence, to obtain higher density at low concentration of deposited probes.

### 3.4. Sensitivity Response of Arrays Made by Microcontact Printing and by Mechanical Spotting

**Figure 7:**
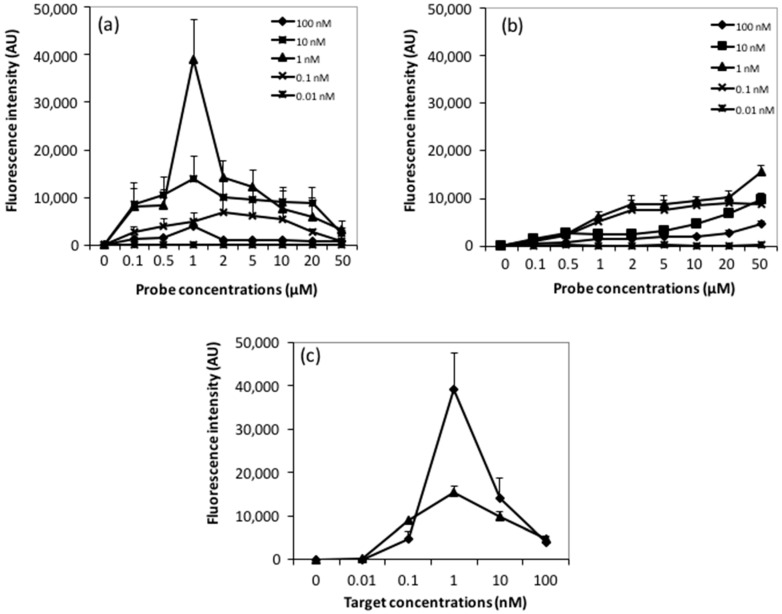
Fluorescence intensities after hybridization at various Cy5-target concentrations (0.01 to 100 nM) on epoxysilane slides as a function of probe concentrations. (**a**) Probes were printed by InnoStamp 40^®^; (**b**) probes were spotted by contact microarrayer; (**c**) fluorescence intensities were recorded for a probe concentration of 1 µM using InnoStamp 40^®^ (diamond) and 50 µM using a conventional spotter (triangle).

The sensitivity response of printed and spotted arrays was evaluated on producing oligoarrays of 20-mer 5′NH_2_-oligonucleotide-probes produced by microcontact printing and mechanical spotting on epoxysilane slides, as described previously. After overnight incubation at room temperature, allowing covalent linkages between NH_2_-oligonucleotides and the epoxysilane surface, low-density microarrays were hybridized overnight at 37 °C with Cy3-labeled 20-mer complementary targets at concentrations ranging from 0.01–100 nM. Results reported in Figure 7 clearly showed an impact of the deposition process on the sensitivity response that is quantitatively expressed by the fluorescence intensity.

**Figure 8:**
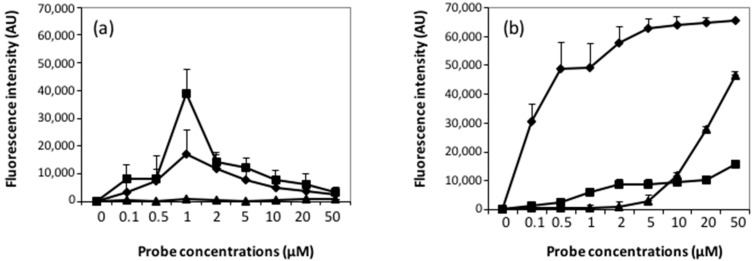
Fluorescence intensities recorded for a target concentration of 1 nM using (**a**) InnoStamp 40^®^ and (**b**) a conventional spotter. Square: epoxysilane slide; diamond: DendriSlide; and triangle: amino-modified slide.

With respect to oligoarrays printed by the InnoStamp 40^®^, the hybridization response showed a bell-shaped behavior at target concentration ranging from 0.1 to 10 nM with an optimal response at 1 nM (Figure 7a and Figure 8a), and a maximal fluorescence intensity obtained at a probe concentration of 1 µM. This probe concentration corresponded to that giving the maximal immobilization efficiency, as inferred from our tests reported above (Figure 6a). In contrast, the mechanical spotting of the probe resulted in fluorescence intensities that increased with the increase of the probe concentration spotted. However, the optimal hybridization response was also found with a target concentration of 1 nM, and the maximal intensity of fluorescence was obtained at 50 µM probe concentration (Figure 7b).

Hence, the sensitivity response, i.e., the lowest concentration of target DNA that gives the highest hybridization signal was the same for both deposition technologies. This data is clearly illustrated in Figure 7c which, furthermore, pointed out the bell-shaped curve of the fluorescence intensity versus the target concentration. Under our assay conditions, we found that 1 nM of a 20-mer oligonucleotide target was the most appropriate concentration that reached the maximal hybridization signal. However, in comparison with the mechanical spotting, this maximal fluorescence signal at this target concentration was obtained at a probe concentration 50-fold lower with the microcontact printing device (Figure 7c).

### 3.5. Effect of the Surface Chemistry on Probe Accessibility and Hybridization Response

In a previous work, we reported on the importance of the surface chemistry in the hybridization response, and showed more specifically that DNA microarrays made with spherical neutral-phosphorus dendrimers-functionalized glass slides (DendriSlides) had a 10-fold higher sensitivity response than those made with other commercially available activated glass slides [30,34]. Since this validation was obtained using the mechanical spotting technology, we wished to re-evaluate this advantage with dendrimer-functionalized glass slide microarrays fabricated by the automated microcontact printing device InnoStamp 40^®^. To this end, we printed and spotted DendriSlides, amino-modified slides and epoxysilane slides with 3′-amino modified 20-mer oligonucleotide probes at concentration ranging from 0.1 µM to 50 µM. After an overnight storage at room temperature, the microarrays were hybridized with a Cy3-labeled 20-mer oligonucleotide targets at the concentration of 1 nM.

Figure 8b reports fluorescence intensity after hybridization with 1 nM labeled complementary targets on spotted microarrays made with the three different types of surface chemistries, which confirmed our earlier report [34]. Indeed, the fluorescence intensity on DendriSlides was already half of its maximum at a probe concentration of 0.1 µM, whereas at this concentration no hybridization of the target was detected on both spotted epoxysilane and amino-modified slides. Increasing the probe concentration from 0.1 to 1 µM only increased by two-fold the fluorescent signal on the spotted DendriSlides, but the signal intensity was already 10-fold higher than with epoxysilane slides at the same spotted probe concentrations. Using epoxysilane slides, the fluorescent signal showed a rather steady augmentation with the increase of the probe concentration, as already reported in Figure 7b. On the other hand, no hybridization signal was measured on the amino-modified slides until the concentration of spotted probes reached 1 µM, which is consistent with extremely low immobilization efficiency for this type of functionalized slide, as we reported in Figure 6d. Above this probe concentration, the fluorescence suddenly raised almost proportionally to the increase of the probe concentrations. This result can be explained when considering that the probe accessibility and the probe density are the limiting factors in target hybridization. Results in Figure 6d support this suggestion since it could be seen that the actual amount of immobilized probes on the amino-modified slides, although remarkably low, increased steadily with the increase of probe concentrations. Taken together, this result further stresses the importance of the surface chemistry in the hybridization response.

At variance with the mechanical spotting method, the hybridization responses obtained with probes printed using InnoStamp 40^®^ on the DendriSlides and on epoxide-functionalized slides were roughly similar, as with both types of surface chemistry. The maximal hybridization response was found at 1 µM of probe concentration. In contrast, no fluorescence signal was measured when the probes were printed on amino-modified slides, which could be explained to a great extent by the extremely low amount of probes retained on this surface.

## 4. Discussion

The manufacturing of low-density microarrays requires the simultaneous deposition of several different oligonucleotide probes at a well-defined position on a solid surface. Currently, these microarrays are performed by using pins or nozzles mounted on commercially available microarrayers [36]. Microcontact printing represents an alternative way to deposit oligonucleotides on solid surfaces [4,27] but, until now, soft lithography techniques are unable to realize a multiplexed printing, which is a severe constraint for the production of DNA microarrays by this technology. To overcome this limitation, we tested the ability of an InnoStamp 40^®^ microcontact printing device and a magnetic MacroStamp^TM^ to produce low-density DNA microarrays. The configuration of the magnetic MacroStamp^TM^ in terms of pillar heights, iron powder concentration, magnetic layer thickness, and magnet motorization in the InnoStamp 40^®^ ensure an accurate control of the applied pressure leading to a reliable and multiplexed deposition of fluorescent oligonucleotide probes. We showed that the magnetic MacroStamp^TM^ composed of 64 pillars, each microstructured at its extremity by a repetition of 50 circular micropatterns of 160 µm diameter, allowed the simultaneous deposition of 64 different molecules in one step at high micropatterned resolutions and without any contamination between oligonucleotide probes inked on adjacent pillars. An advantage of the thin magnetic layer (5 mm) is that the MacroStamp^TM^ maintains a high flexibility, ensuring the contact of all microstructured pillars with the solid surfaces. In opposition to the mechanical spotting from which individual oligonucleotides are sequentially spotted in duplicate or triplicate, the printing configuration of the MacroStamp^TM^ results in much greater replication of the same oligonucleotide probe, which can allow a statistical analysis of the fluorescent signal arising either from the deposition of the probe (qualification of the printing process) or after hybridization of the probe with the labeled target. Advantageously, a MacroStamp^TM^ could be reused up to 10 times without loss of its oligonucleotide transfer properties by a simple and fast ethanol washing, allowing the deposition of around 32,000 spots (64 × 10 × 50) using a single MacroStamp^TM^. The remarkable efficiency and rapidity of the washing step combined with the absence of variability between MacroStamp^TM^ batches and the multiplexed deposition of oligonucleotide probes make the InnoStamp 40^®^/MacroStamp^TM^ a reliable platform to produce low-density DNA microarrays by the microcontact printing technology.

An important parameter in microarray fabrication processes is the immobilization efficiency of oligonucleotide probes. Since the probes’ deposition process on a solid support by microcontact printing tools is carried out under dry conditions, whereas the probes are spotted as liquid droplets by metal pins from conventional spotting tools, we were interested in examining whether both technologies led to similar amounts of deposited oligonucleotide probes. After an hybridization-mimicking step (hybridization conditions without complementary target) on epoxysilane- (covalent bonding) or aminosilane- (electrostatic interactions) modified glass slide functionalized with a doubly 5′-amino-3′Cy3 oligonucleotide, the amount of probes printed on both slides by microcontact printing was roughly independent to the initial concentration of probes. One possible explanation for this observation is that the deposition using microcontact printing leads to the transfer of a uniform layer of oligonucleotides from the PDMS stamp to the slide surfaces. During the inking step all available sites on the PDMS stamp surface are probably occupied even at the lowest probe concentration of 0.01 µM. Using the contact microarrayer, the fluorescence intensities reached saturation at a probe concentration of 0.5 µM on epoxysilane slide and of 50 µM on aminosilane slide. We observed that the microcontact printing technology results in lower immobilization efficiency than the mechanical spotting method.

One of the most relevant parameters in the DNA microarray field is the sensitivity of the interaction between the probe and target DNA molecules, which can be defined as the lowest concentration of target DNA that gives a measurable signal intensity (commonly defined as at least three times the intensity of the background level) when it hybridizes with its complementary probe. This sensitivity will depend on several parameters, including probe and target concentrations, probe accessibility, as well as the process of probe depositions on the slide surface [37]. In the case of oligoarrays produced by microcontact printing, a bell-shaped curve was obtained after hybridization of oligonucleotide targets with an optimal response at unexpected low probe and target concentrations respectively 1 µM and 1 nM. This bell-shaped behavior is, at first glance, surprising because, at least in the case of DNA microarrays fabricated by the conventional spotting method, it is assumed that the probes are in excess of the amount of targets delivered on the arrays. Since an even higher intensity signal was obtained with microcontact-printed oligonucleotides using a 50-fold lower concentration of probes (Figure 7c), it is unlikely that this bell-shaped hybridization response is due to probe densities. However, this result suggests that the microcontact printing method leads to uniform printed pattern features without the typical rings observed in spotted droplets due to buffer evaporation (coffee stain effect [38]) and renders the probe more accessible to their targets compared to conventional spotting. On the other hand, the bell-shaped hybridization response could be explained by the occurrence of auto-quenching effects at high probe and target densities, which reduces the fluorescence signal. It has been pointed out that the requirement for an enhancement of the hybridization rate involves a surface sparsely covered with oligonucleotide probes, which interact with labeled targets of high strength and selectivity [39]. Peterson et al. [40] also reported that, at low probe densities, almost 100% of the probes hybridize, while at high probe densities only 10% of the probes hybridize and the kinetics of binding is slow. Probe density may result in steric effects [41] and could affect the formation efficiency and kinetics of target capture.

## 5. Conclusions

In this work, we report on the use of an automated microcontact printing device employing soft-lithography technology to produce low-density multiplexed DNA microarrays. This multiplexed microcontact printing was made possible by the use of a MacroStamp^TM^ that bears 64 individual pillars each mounted with 50 circular micropatterns (spots) of 160 µm diameter at 320 µm pitch. A fast and efficient washing step in 96% ethanol made the MacroStamp^TM^ reliable and reusable. The low-density microarrays printed on either epoxysilane (Nexterion E) and dendrimer-functionalized slides (DendriSlides) turned out to show excellent hybridization responses with complementary sequences at unusually low probe and target concentrations (respectively, 1 µM and 1 nM). In addition, our comparative analysis of microarrays fabricated by mechanical spotting and by the automated microprinting method highlights the importance of the accessibility rather than the absolute density of the probes in the hybridization response and provides the basis for the latter technology as a new potential platform in the fabrication of low-density DNA microarrays.
